# Impact of wound closure on fibular donor-site morbidity: a meta-analysis

**DOI:** 10.1186/s12893-019-0545-1

**Published:** 2019-07-05

**Authors:** Hui Fang, Fayu Liu, Changfu Sun, Pai Pang

**Affiliations:** Department of Oromaxillofacial-Head and Neck Surgery, Oral Maxillofacial Surgery, School of Stomatology, China Medical University, 117 Nanjing Bei Jie, Heping, Shenyang, Liaoning 110002 People’s Republic of China

**Keywords:** Free fibular flap, Donor-site morbidity, Primary closure, Skin graft

## Abstract

**Background:**

Vascularized free fibular flaps have been the “workhorses” for reconstruction of many kinds of bone defects. Nevertheless, there is no consensus regarding the optimal wound closure method for fibular donor sites. This study aimed to compare prognostic outcomes of primarily closures (PC) and skin grafts (SG) for fibular donor sites.

**Methods:**

Studies regarding donor-site outcomes of PC versus SG in patients undergoing free fibular flap procedures were included. Two authors individually searched PubMed, Web of Science, EMBASE, Cochrane Library and clinicaltrials.gov up to February 2019, extracted the data and assessed quality of each selected article. Ultimately, The incidences of donor-site morbidities were evaluated.

**Results:**

Five studies with a total of 119 patients were included in our analysis. No significant differences were found with respect to the rates of donor-site problems between the PC and SG groups.

**Conclusions:**

Fibular flap patients undergoing PC and SG wound closures may have similar donor-site outcomes. Additional large-scale studies are necessary to draw a solid conclusion.

**Electronic supplementary material:**

The online version of this article (10.1186/s12893-019-0545-1) contains supplementary material, which is available to authorized users.

## Background

Since it was first introduced for extremity reconstruction, the vascularized free fibular flap has been widely used for tibia, radius, mandible and many kinds of bone reconstructions [1–3] because of its adequate bone length, reliable blood supply and flexible application to both bone and soft tissue reconstructions [4, 5]. After harvesting the donor site, early complications include wound dehiscence, infection, and loss of skin graft; late complications include permanent pain, ankle instability and restriction of movement [6–8]. Most of the existing studies of fibular flap donor-site morbidities have small sample sizes and lack consistency. Guidelines for prevention, systematic evaluation and treatment of fibular donor-site morbidities is needed.

The wound of donor-site could be closed primarily or may be covered with a skin graft. It is generally accepted that using a skin graft may leave an obvious scar, as well as damaging to the second donor-site and causing skin graft necrosis [9–11]. However, directly closing the wound under tension presents a larger possibility of wound healing problems and compartment syndrome [12]. Although many new methods and devices have been reported to facilitate donor-site wound healing and to reduce the incidence of complications, the method that provides better outcome remains controversial.

In this study, we analyzed five studies comparing donor-site outcomes of free fibular flaps with primary closure (PC) or a skin graft (SG) to guide clinical decision-making.

## Methods

This meta-analysis was performed in accordance with the Preferred Reporting Items for Systematic Reviews and Meta-Analyses (PRISMA) reporting guidelines for the conduct of meta-analysis of intervention trials [13].

### Search strategy

PubMed, Web of Science, EMBASE, Cochrane Library and clinicaltrials.gov were searched for studies regarding donor-site morbidity of fibular flap procedures up to February 2019. Articles published in English and Chinese including the following keywords were included: (Donor Site OR Donor Sites OR Donor-Site OR Donor Site, Transplant OR Donor Sites, Transplant OR Site, Transplant Donor OR Sites, Transplant Donor OR Transplant Donor Sites) AND (Fibula OR Fibulas OR Fibular flap OR Fibula flap OR Fibula graft) AND (Grafting, Skin OR Graftings, Skin OR Skin Grafting OR Skin Graftings OR Dermatoplasty OR Dermatoplasties OR Transplantation, Skin OR Skin Transplantations OR Transplantations, Skin). The list of references of related articles was manually searched for missing papers.

### Inclusion/exclusion criteria

We used Endnote X7 software to manage and delete duplicate articles. When a study team published a series of articles, only the latest study was included.

Inclusion criteria:

1. Studies comparing donor-site morbidity of fibular free flaps with a primary closure or skin graft

Exclusion criteria:

1. Studies providing data for a single method of wound closure without comparison.
2. Studies using other methods or device closures of the donor-site wound.
3. Studies not providing sufficient data regarding patient number or rates in the PC and SG group.
4. Studies harvesting more than one lower limb free flap.
5. Studies reporting only necrosis of skin graft without comprehensive comparisons.
6. Studies comparing PC or SG with other treatments.
7. Meta-analyses, reviews, letters, meeting abstracts, case reports and editorials.

### Data extraction and quality assessment

Two authors (FH and PP) independently conducted data extraction and quality assessment. The result was crosschecked and controversies were discussed with another author (SCF). Basic information such as first author, publication year, country, recipient site and other relevant data were extracted. And only clinical cohort studies were included. We assessed the study quality with the Newcastle-Ottawa Quality Assessment Scale. A maximum of 9 stars can award to each study, and studies with more than 5 stars were considered of good quality for further research [14].

### Statistical analysis

We used STATA 13.0 (Stata Co., College Station, TX) for statistical analysis, and relative risks (RR) and 95% confidence intervals (CI) to calculate the associations between PC and SG groups. Heterogeneity was evaluated by Chi-squared-based Q test and I^2^. If *P* > 0.10 and I^2^ < 50%, we adopted a fixed effects model with Mantel-Haenszel calculation method. If *P* < 0.10 or 50% < I^2^ < 70%, we adopted a random effects model with D-L method. A z-test was also used with *P* < 0.05 was considered statistically significant. We performed sensitivity analysis to evaluate statistical stability and Begg’s test and Egger’s liner regression to evaluate publication bias.

## Results

### Studies and population

**Fig. 1:**
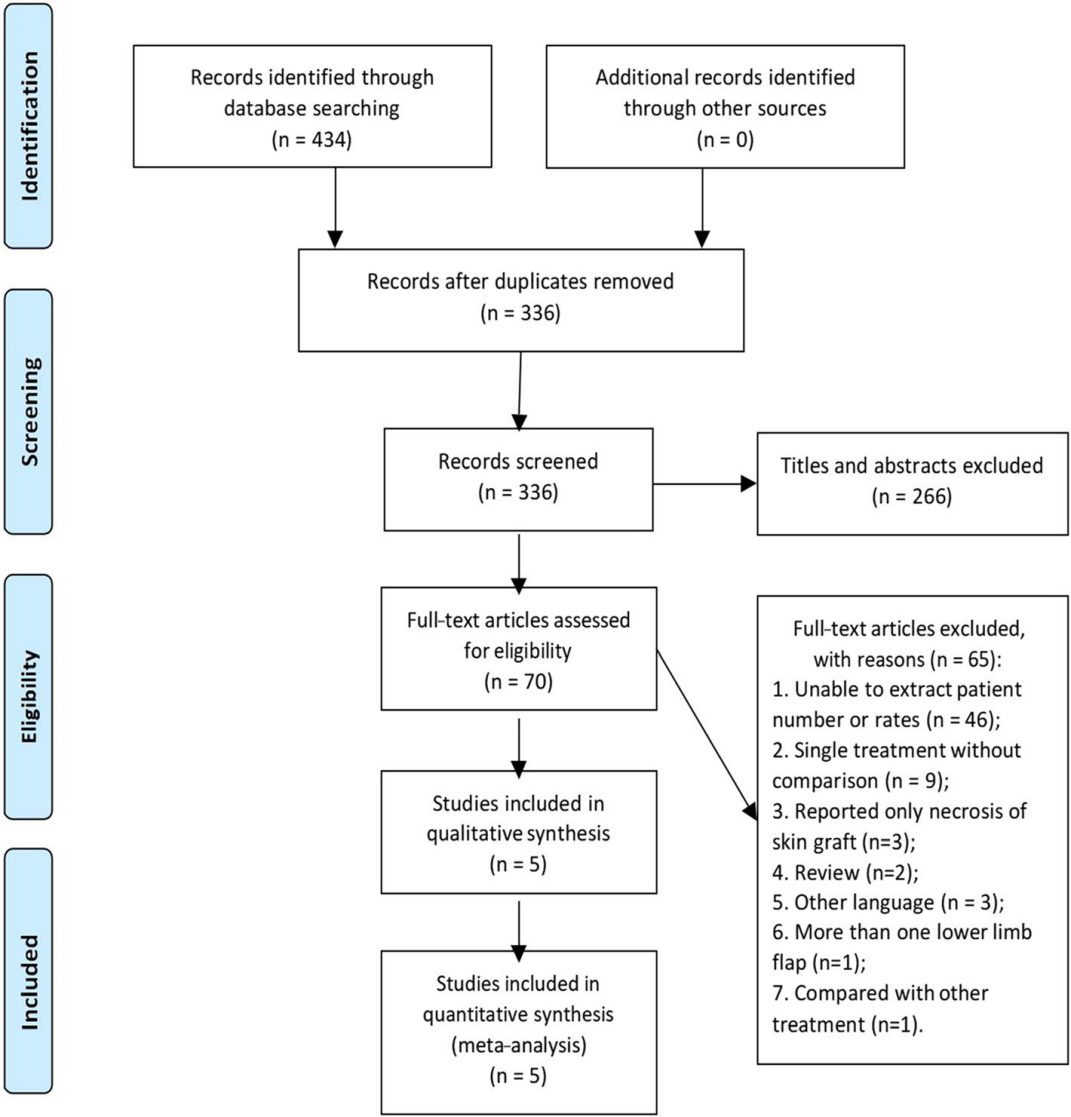
PRISMA flow diagram for the study selection process

There were 434 records identified after searching the database and reviewing relevant articles. Three hundred and thirty-six articles were left after deleting the duplications. Then, title and abstract were screened and 70 studies were retained for full text evaluation. In this step 46 articles did not provide number or rates of patients undergoing PC or SG. Nine studies included patients undergoing only PC or SG without comparison. Three studies only reported necrosis of skin grafts but did not generally evaluate donor-site morbidity for both groups. Two reviews and three papers published in French, Japanese and Spanish were excluded. One study included patients undergoing anterolateral thigh flap and fibular free flap at the same time. One study compared outcomes of two different kinds of skin grafts. Therefore, five studies with 119 participants were included for data synthesis (Table 1). We followed the PRISMA guidelines and the study selection procedure is illustrated in a PRISMA flow diagram (Fig. 1).

**Table 1:** The characteristics of the studies included in this meta-analysis

First author	Year of Publication	Country	Study size (PC/SG)	Recipient site
Hidalgo, D. A	1989	America	10/2	Mandible
Jupiter, J. B	1997	America	3/6	Radius
Shindo, M	2000	America	26/27	Head and neck defect
Roan, T. L	2013	Taiwan	4/6	Head and neck defect
Akashi, M	2016	Japan	24/11	Head and neck defect

All included articles were published in English. Three studies were conducted in America, one was in Taiwan, and one was in Japan. All 119 included patients underwent free fibular flap harvesting for head and neck or radius defects. Sixty-seven donor sites were closed directly and 52 patients underwent skin graft transplantation. The quality assessment results are presented in Table 2. All studies included patients with good representativeness, good comparability between groups and clear outcomes data.

**Table 2:** Result of literature quality assessment according to the Newcastle-Ottawa quality Assessment Scale

Study	Selection	Comparability	Outcome
Hidalgo 1989	☆☆☆☆	☆	☆☆
Jupiter 1997	☆☆☆☆	☆☆	☆☆
Shindo 2000	☆☆☆☆	☆	☆☆
Roan 2013	☆☆☆☆	☆☆	☆☆
Akashi 2016	☆☆☆☆	☆	☆☆

### Meta-analysis of donor-site morbidity of fibular free flap with PC versus SG

**Fig. 2:**
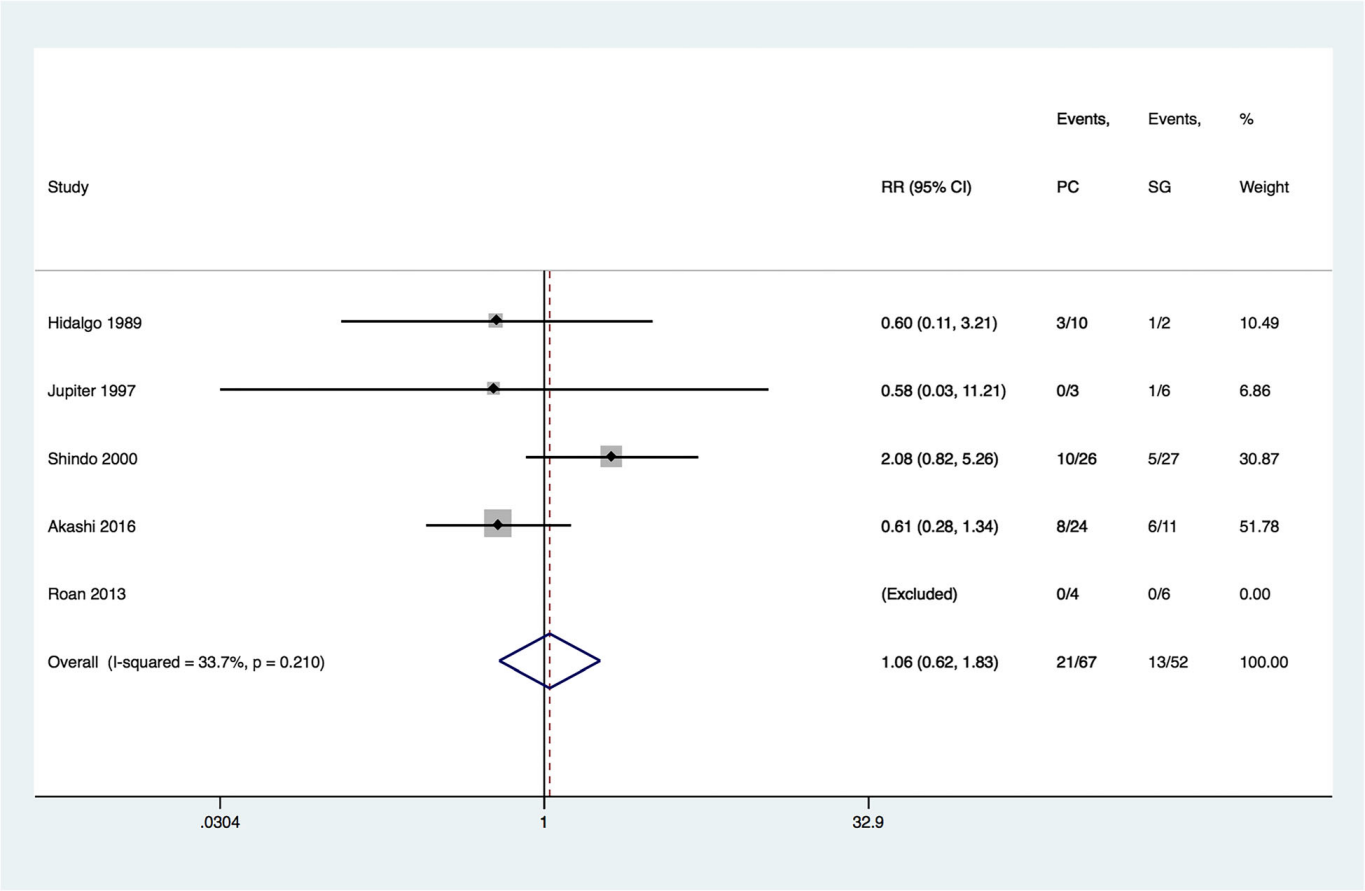
Forest plots comparing PC versus SG

Because of the double-zero events in Roan’s research [15], 4 studies were finally eligible for data synthesis. A heterogeneity test showed no significant heterogeneity between studies (I^2^ = 33.7%, P_Q-test_ = 0.210); therefore, a fixed effects model with the Mantel-Haenszel method was used. The meta-analysis suggested that there were no substantial differences in incidences of donor-site morbidities between PC and SG groups (RR = 1.061, 95% CI 0.616–1.826, P _z-test_ = 0.832, Fig. 2). Sensitivity analysis and publication bias test were performed but the result was not shown due to the small number of included studies (Additional file 1).

## Discussion

Since Taylor first introduced the fibular free flap procedure to reconstruct tibial defects in 1975, this procedure has been considered the “workhorse” for bone reconstruction with minor donor-site morbidity [16, 17]. However, in 2012, a systematic review focused on fibular flap donor-site morbidity showed that, from wound healing problems to functional impairment, the incidence of postoperative donor-site complication rates ranged from 1.07 to 19.0% [6]. In Momoh’s cohort study that included 157 fibular flap patients, this number was 31.2% [18]. By using balance and gait tests, Lin et al. found that 14% of fibular flap patients had pain after prolonged walking, 28% had difficulty squatting, and 14% patients had minimal paresthesias in the donor-leg [5]. Similarly, Xu found that values of isokinetic testing on the donor-side ankle joint showed a significant decrease 1 year postoperatively, and plantar center pressure shifted to the heel on the donor side at 6 months after surgery [7].

With respect to donor-site wound closure of fibular flaps, there has been an ambiguous definition. Some agree that a defect width of donor site less than 6 cm could be closed directly [19], whereas some believe that primary closure is possible only when a defect narrower than 4 cm [20]. In the 5 articles that are included in our research, three studies gave the width of fibular flaps of 54 patients (31 PC and 23 SG patients). The average flap width of these PC patients are 5.71 cm, the average flap width of SG patients are 6.41 cm. It is generally acknowledged that closing the donor-site with a skin graft would consume more time, leave a noticeable scar and cause damage to the secondary donor site of the skin graft [9, 10]; however, direct closure under tension may result in wound healing problems or even compartment syndrome [12, 21]. According to Shindo’s study, patients with a donor-site defect as narrow as 2.5 cm could have wound complications after direct wound closure [12]. This suggests that the width of the donor-site defect alone is not the determining factor regarding whether to close the defect primarily. A tension evaluation of the donor-site before closing the wound may largely reduce the incidence of healing problems.

Furthermore, many other factors have important impacts on donor-site healing. Li et al. used bivariate correlation analysis to assess the risk factors for early and late donor-site complications of free fibula flaps [22]. They found that harvested fibular length, operation time and follow-up time were important factors for late donor-site morbidity, whereas no domain showed a statistically significant association. In Shindo’s evaluation, heavy smokers had a significantly increased incidence of donor-site complications [12]. He also speculated that time of muscle edema and skin paddle location were two other important variables affecting donor-site restoration [12].

To reduce the incidence of donor-site morbidities, many new devices and surgical techniques have been used in wound closure. Berend et al. used a local boat-shaped full-thickness skin graft to close the donor-site wound, thus avoiding secondary donor-site damage [23]. Sharma introduced a local propeller flap for the closure of the fibular flap skin donor site instead of a skin graft [24]. Fry et al. used creation of a lattice to aid partial closure to achieve secondary intention healing [25]. All of these studies presented good treatment effects and increased donor-site prognoses. Currently, it is agreed by many surgeons that late donor-site morbidities of fibular flaps have higher incidences than do early complications [16, 22, 26]. Therefore, a sufficient long-term follow-up is necessary to obtain a comprehensive observation of donor-site morbidity, a feature that is absent in many existing studies.

## Conclusions

We analyzed and synthesized data from five studies comparing fibular flap donor-site outcomes of skin graft transplantation versus primary closure. The meta-analysis showed that there were no significant differences in donor-site morbidity rates between primary closure and skin graft groups. In consideration of the limited number of patients in this study, additional large-scale studies are necessary to draw a solid conclusion.

## Additional file

Additional file 1: Sensitivity and publication bias analysis. A. Sensitivity analysis comparing PC versus SG; B.Begg’s funnel plot of PC versus SG; C. Egger’s liner regression of PC versus SG. (JPG 254 kb)

## Data Availability

The datasets generated and analysed during the current study are available in the Figshare repository with a DOI of https://figshare.com/s/6b74689e00cf5b5263a8, and available from the corresponding authors.

## Abbreviations

CI: Confidence interval
PC: Primarily closure
PRISMA: Preferred Reporting Items for Systematic Reviews and Meta-Analyses
RR: Relative risk
SG: Skin graft
