# Supplementary figures and images for: Impact of wound closure on fibular donor-site morbidity: a meta-analysis
Source: BMC Surg. 2019 Jul 5;19:81. doi: 10.1186/s12893-019-0545-1 (PMC6612155; doi:10.1186/s12893-019-0545-1)

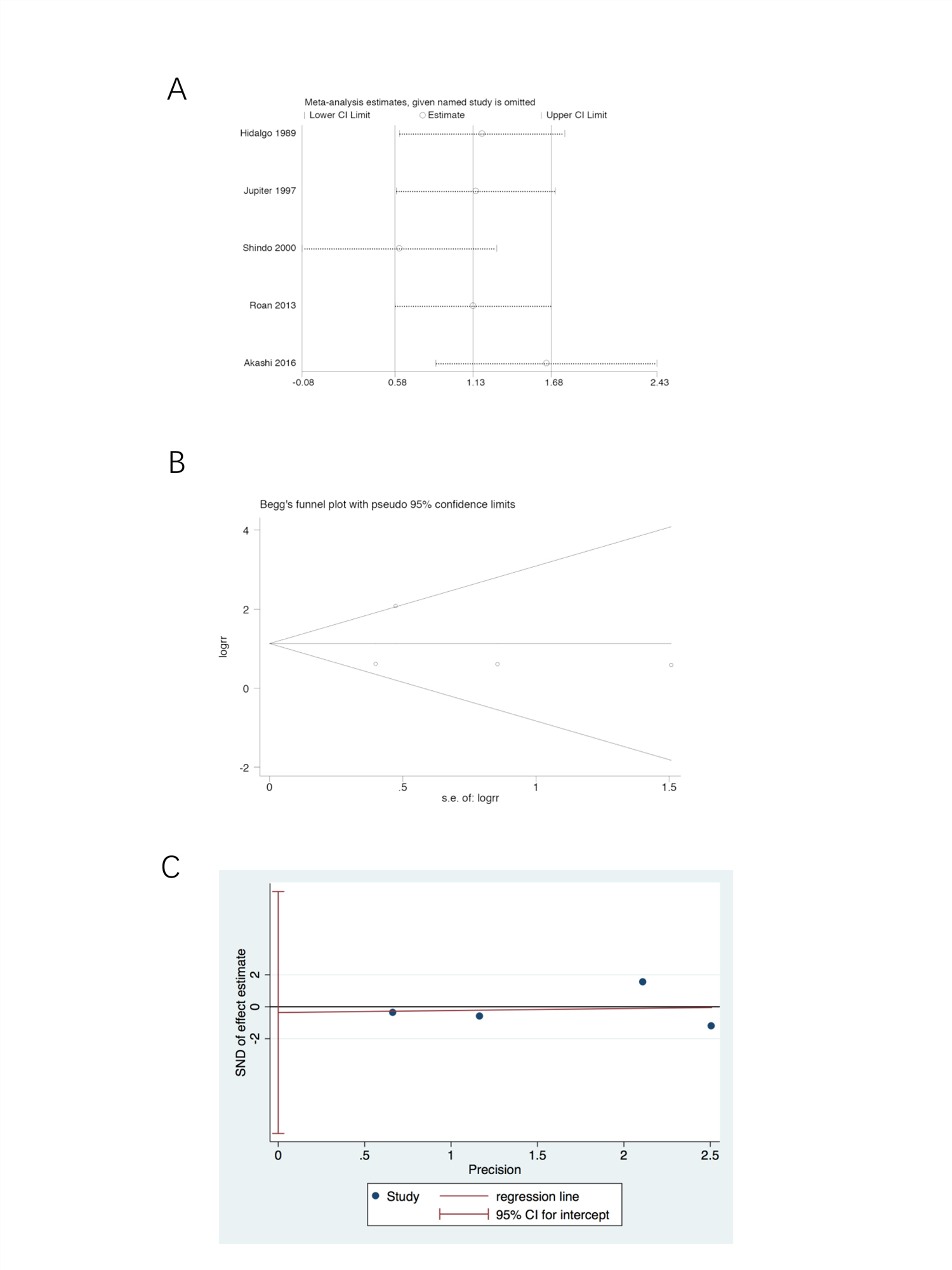

Supplement: Supplementary file 1 — Sensitivity and publication bias analysis. A. Sensitivity analysis comparing PC versus SG; B.Begg’s funnel plot of PC versus SG; C. Egger’s liner regression of PC versus SG. (JPG 254 kb) [file 12893_2019_545_MOESM1_ESM.jpg]
